# *Hymenolepis nana* antigens alleviate ulcerative colitis by promoting intestinal stem cell proliferation and differentiation via AhR/IL-22 signaling pathway

**DOI:** 10.1371/journal.pntd.0012714

**Published:** 2024-12-12

**Authors:** Xuanyin Cui, Yi Cheng, Hongyan Wang, Xiaomao Li, Jinfu Li, Ke Zhang, Rong Mou

**Affiliations:** Guizhou Key Laboratory of Microbio and Infectious Disease Prevention & Control / The Key and Characteristic Laboratory of Modern Pathogenicity Biology, Department of Human Parasitology, School of Basic Medicine, Guizhou Medical University, Guiyang, China; Salk Institute for Biological Studies, UNITED STATES OF AMERICA

## Abstract

Ulcerative colitis (UC) is a chronic inflammatory bowel disease with an unknown etiology and is difficult to treat. Studies have shown that some helminths and their associated products have therapeutic potential in controlling or preventing inflammatory diseases. This study is to investigate the mitigation effects of *Hymenolepis nana* antigens (HnAg) on the UC model. HnAg significantly improved the disease activity index, colon length, and colonic pathological damage in mice with dextran sulfate sodium (DSS)-induced colitis. HnAg intervention could protect the number of goblet cells and enhance the expression of tight junction proteins and mucins, thereby improving intestinal barrier integrity. HnAg attenuated small intestinal organoid damage and stimulated intestinal stem cells proliferation in a DSS-induced mouse organoid inflammation model. The protective mechanism of HnAg might be related to the activation of the aryl hydrocarbon receptor (AhR)/IL-22 signaling pathway, which regulates intestinal barrier function and promotes the proliferation and differentiation of intestinal stem cells. In conclusion, HnAg has a therapeutic effect on UC mice. Our study provides a new approach for alleviating UC by *Hymenolepis nana* and its associated products.

## Author summary

Ulcerative colitis (UC) is an immune-mediated chronic inflammatory disease. Currently, commonly used drugs such as aminosalicylates and glucocorticosteroids are difficult to treat and have many adverse effects. In recent years, there has been a growing interest in utilizing the immunomodulatory abilities of worms to develop new therapies for the treatment of inflammatory and allergic diseases. *Hymenolepis nana* is a zoonotic parasitic worm that parasitizes the small intestines of humans and rodents and generally does not cause serious pathologic damage to their hosts, making it a potential class of intestinal parasites in helminthic therapy. In this study, we investigated the mitigating effect of *Hymenolepis nana* antigens (HnAg) on UC in mice from the perspective of maintaining intestinal mucosal barrier integrity. The results showed that HnAg alleviated UC in mice by activating the aryl hydrocarbon receptor (AhR)/IL-22 signaling pathway, promoting the proliferation and differentiation of intestinal stem cells, and modulating the integrity of the intestinal mucosal barrier. Meanwhile, HnAg attenuated DSS-induced inflammatory damage in mouse small intestinal organoid tissues and promoted intestinal stem cells proliferation. Suggesting that *Hymenolepis nana* and its related products may become a new option for alleviating UC.

## 1. Introduction

Ulcerative colitis (UC) is an immune-mediated chronic inflammatory disease that predominantly affects the mucosal and submucosal layers of the colon [1]. Clinically, it manifests as severe gastrointestinal symptoms such as abdominal pain, diarrhea, and bloody stools, a long and recurring disease with multiple complications [2]. Mucosal healing is recognized as the golden standard to induce long-term remission, the goal of clinical therapy for UC [3]. The core component of the intestinal mucus layer is mucin 2 (MUC2), which is secreted by goblet cells and helps to separate the gut microbiota from the mucosal epithelial monolayer [4]. Reduced production of mucins, along with decreased levels of tight junction proteins such as Claudin-1, Occludin, and ZO-1, significantly impairs the barrier function of the intestine [5].

Mucosal healing is accomplished by regenerating and repairing the intestinal epithelium [6]. Therefore, repairing damaged intestinal epithelium is crucial to prevent sustained intestinal mucosal damage caused by UC. Intestinal epithelial cells undergo rapid renewal every 3–5 days [7]. The regeneration and reconstruction of the intestinal epithelium are primarily dependent on interleukin-22 (IL-22). IL-22 can strengthen the intestinal barrier by promoting tight junctions [8] and maintain intestinal homeostasis by promoting the regeneration of intestinal stem cells (ISCs) [9]. Therefore, promoting the production of IL-22 is essential for epithelial repair. In the gut, the aryl hydrocarbon receptor (AhR) plays an active role in maintaining normal cellular physiological functions and is closely linked to intestinal immune responses, intestinal barrier function, inflammatory responses, and the intestinal microenvironment. Cytochrome P450 1A1 (CYP1A1) is a direct downstream signaling molecule of the AhR, and the expression level of CYP1A1 can typically be associated with the activation status of AhR [10]. AhR also promotes the production of IL-22 by group 3 innate lymphoid cells (ILC3) [9]. In addition, AhR mainly relies on IL-22 to suppress gastrointestinal inflammation. Activation of AhR upregulates IL-22 to regulate chronic intestinal inflammation [11].

Currently, the treatment of UC primarily relies on aminosalicylates, glucocorticoids, immunosuppressants, and biological inhibitors, but there are shortcomings such as difficulty in cure and many adverse reactions [12]. Therefore, finding effective treatments with minimal side effects has become a pressing scientific issue. In recent years, there has been increasing interest in utilizing the immunoregulatory capabilities of helminths to develop new therapies for the treatment of inflammatory and allergic diseases [13]. Epidemiological evidence from the hygiene hypothesis of IBD suggests that helminth infections can modulate certain immune-mediated diseases [14]. Studies of the effects of the intestinal epithelium have typically used the intestinal epithelial cell lines IEC-6 and Caco-2 cells [15], which do not fully mimic the “real” situation in the intestine. The new approach that ISC-containing intestinal organoids can proliferate and differentiate into all intestinal epithelial cell lines has been applied to the study of inflammatory bowel disease [16]. *Hymenolepis nana* (*H*. *nana*) is a zoonotic parasitic helminth that inhabits the small intestine of humans and rodents. Research on *H*. *nana* has been conducted in epidemiology, clinical manifestations, and treatment [17]. The crude antigens and excretory-secretory products of *Hymenolepis diminuta* (*H*. *diminuta*) have been shown to alleviate UC, which is a close species of *H*. *nana* [18,19]. However, there have been no reports on the role of *H*. *nana* in alleviating inflammatory diseases. Furthermore, few studies have reported on the role of intestinal helminths or their derived products in promoting epithelial regeneration and repair from the perspective of ISCs in UC.

In the current study, we evaluated the therapeutic effects of HnAg on UC by using a dextran sulfate sodium (DSS)-induced UC mouse model and a DSS-induced inflammation model of intestinal-like organoids in mice. We investigated that HnAg promoted the proliferation and differentiation of ISCs through the AhR/IL-22 signaling pathway to achieve protection of damaged intestinal mucosa.

## 2. Materials and methods

### Ethics statement

All animal experiments of the current study were approved by the Medical Ethics Committee of Guizhou Medical University (approve Nos. 2100346, 2100347).

### 2.1 Reagents

DAB staining kit (ZLI-9018, zsbio), dextran sulfate sodium (DSS, MW: 36,000–50,000 Da, MP), carmine (1390-65-4, Macklin), and salicyzosulfapyridine (SASP, 599-79-1, Aladdin) were offered by Guiyang Jingong Technology Co (Guiyang, China). Masson’s Trichrome Stain Kit (G1343), Hematoxylin-Eosin Stain Kit (G1120), EDTA antigen retrieval solution (C1034), Fecal Occult Blood Kit (BC8270), and DAPI solution (C0065) were offered by Solarbio (Beijing, China). AB-PAS Stain Kit (R22021) was obtained from Saint-Bio (Shanghai, China). RT-qPCR Reverse Transcription Kit (11141ES60), SYBR RT-qPCR Kit (11201ES08), RIPA lysis buffer (20101ES60), BCA Protein Quantification Kit (20201ES76), TRIeasy Total RNA Extraction Reagent (10606ES60), MolPure Cell/Tissue DNA Kit (18700ES50), and Super enhanced chemiluminescence (ECL) Detection Reagent (36208ES60) were provided by Yesen (Shanghai, China). Goat serum (abs933) was purchased from Absin (Shanghai, China). Mouse Intestinal Organoid Kit (K2001-MI) was offered by bioGenous (Jiangsu China). Basement Membrane Matrix GFR (HY-K6004) was obtained from MCE (Shanghai, China).

### 2.2 Animal experiments

Primary antibodies for western blotting or immunohistochemistry: ZO-1 (21773-1-AP), β-tubulin (10094-1-AP), and Occludin (66378-1-IG) were offered by Proteintech (Wuhan, China), for Lgr5 (R380973) was obtained from ZenBio (Chengdu, China), for IL-22 (ER1803-85) was provided by HuaBio (Hangzhou, China), for CYP1A1 (sc-101828) was offered by Santa Cruz Biotechnology (Dallas, TX, USA), and GAPDH (AF7021) was purchased from Affinity (Jiangsu, China). Secondary antibodies for western blotting and immunohistochemistry (HY-P8001 and HY-P8004) were purchased from MCE (Shanghai, China). Primary antibodies for immunofluorescence or immunohistochemistry: MUC2 (ab272692), Dclk1 (ab31704), and ChgA (ab283265) were obtained from Abcam (Cambridge, UK), for Lgr5 (DF2816) was purchased from Affinity (Jiangsu, China). PCNA (PA5-27214, MA5-11358) and fluorescent secondary antibody anti-rabbit 488 (A21206) were offered by Thermo Fisher Scientific (Waltham, MA, USA).

#### 2.2.1 The hamsters and the acquisition of serum from *H*. *nana* infected hamsters

The 4–6 weeks, weighing approximately 16.7 ± 2.9 grams (g), and female hamsters (*Phodopus sungorus*) (*n* = 40) were purchased from the pet market in Nanming District, Guiyang, China in December 2023 (S1A Fig). Hamsters were sacrificed under anesthesia to obtain intestinal parasites, of which twenty-eight were infected by parasites with an infection rate of 70% (28/40). A few parasites were randomly selected and cut with a scissor to obtain eggs. In addition, five parasites were randomly selected from those obtained and stained with carbolic acid red (configured with carmine), eggs and stained adults were observed using an upright fluorescence microscope (Eclipse 80i, Nikon Ltd, Japan). A few parasites were re-selected and worm DNA was extracted using a tissue DNA Kit, and PCR amplification of the *COX-I* gene of *H*. *nana* followed by agarose gel electrophoresis. The parasites obtained from hamsters were identified as *H*. *nana* (S1B–S1F Fig). The *COX-I* primer sequence used in this process is detailed in S1 Table. For serum from *H*. *nana* infected hamsters, blood samples were obtained from the eyeballs of *H*. *nana* infected hamsters, stored at room temperature for 2 h, centrifuged at 10,000 x g for 10 min, then aspirated the serum and stored at -80°C.

#### 2.2.2 The C57BL/6J mice experiments

The specific-pathogen-free (SPF), 6–8 weeks, weighing approximately 22.0 ± 2.0 g, and female C57BL/6J mice (*n* = 40), were purchased from the Experimental Animal Center of Guizhou Medical University (SCXK (Jing) 2019–0010). The mice were housed in standard laboratory conditions, free of parasitic contamination, maintained at a temperature range of 20–22°C, and subjected to a controlled 12-h light/dark cycle. Following a seven-day period of adaptive feeding, the following experiments were conducted.

SASP is a drug known to be effective in treating UC, so it was chosen as a positive drug intervention control for this experiment [20]. The UC animal model and the intervention approach were based on the pieces of literature [19,21–23]. In brief, the animals were randomly assigned to four groups: a Control (Ctrl) group (*n* = 10), a DSS group (*n* = 10), a DSS + HnAg group (*n* = 10), and a DSS + SASP group (*n* = 10). To establish the DSS group as an acute UC animal model, mice were provided with sterilized 4% (m/v) DSS dissolved in H_2_O for 7 consecutive days. Additionally, mice in the DSS + HnAg group and DSS + SASP group were similarly fed with sterilized 4% (m/v) DSS dissolved in H_2_O for 7 days. However, mice in the DSS + HnAg group received intraperitoneal injections of 500 μg/day of HnAg, while mice in the DSS + SASP group were orally administered 250 mg/kg/day of SASP. On the 8^th^ day, all mice were anesthetized and euthanized.

#### 2.2.3 Mouse small intestine organoid experiments

**2.2.3.1 Crypt isolation and intestinal organoid culture.** Small Intestinal organoids were obtained from the intestines of 6 to 8-week-old C57BL/6J mice, as described in the literature [24]. Briefly, mice were anesthetized and executed to obtain small intestines, which were dissected and cut into pieces, then washed several times with pre-cooled DPBS until the supernatant became clear. The small intestine pieces were then incubated in DPBS containing 5 mM EDTA for 20 min on ice. Subsequently, the crypt portion was isolated by blowing the tissue with a spiking gun and passing it through a 70 μm cell strainer, and centrifuging at 200 x g for 5 min. To remove single cells, the crude crypts obtained were purified by centrifugation at 150 × g for 3 min. The crypt suspension was mixed with an equal volume of Matrigel and then seeded into 24-well culture plates at 30 μl per well. The plates were incubated in an incubator at 37°C, 5% CO_2_ for 20 min. After Matrigel solidification, 500 μl of mouse small intestine organoid complete medium was added and the medium was completely changed every two days.

**2.2.3.2 Modeling and co-culture of small intestinal organoid inflammation.** Inflammation modeling was carried out according to the literature [25]. Briefly, after three days of culture of the organoids, they were divided into Ctrl, DSS, and DSS+HnAg (low, medium, and high) groups, and DSS (4 μM) was added and cultured for 24 h. Then 2 μg/ml, 4 μg/ml, and 8 μg/ml of HnAg were added to continue the culture for 24 h, and the emergence of the organoids was observed under an inverted microscope (DS-Ri2, Nikon Ltd, Japan). Small intestinal organoids were collected and preserved at -80°C for subsequent experiments.

### 2.3 The extraction and identification of adult *H*. *nana* antigens (HnAg)

After obtaining *H*. *nana* from hamsters, the worms underwent multiple washes with sterile PBS before being homogenized using a hand-held device (MT-13K-L, MIULAB, Hangzhou, China). The homogenate samples were then centrifuged at 10,000 x g for 10 min, and the supernatant was carefully collected. After protein concentration was measured by the BCA assay, the supernatant was stored at -80°C for the following assays. Meanwhile, the number and molecular size of proteins contained in HnAg were evaluated by immunoblotting. Briefly, 5% stacking gel and 10% resolving gel were meticulously prepared. Each well of the gel was loaded with 15 μg of protein. After electrophoresis, the protein samples were transferred to a PVDF membrane, which was subsequently blocked with 5% skim milk powder to minimize nonspecific binding. Primary antibody (serum from *H*. *nana* infected hamster, diluted to 1:50) was applied to the membrane and incubated overnight. At the next day, an anti-mouse secondary antibody (1:10,000) was added and incubated for 1 h. Finally, Super ECL Detection Reagent was utilized for sensitive detection of the protein.

### 2.4 Disease activity index (DAI) score

The body weight, shape of feces, and fecal occult blood of the mice were measured daily. The DAI was calculated using the following formula:

DAI = (weight change score + loose stools score + bloody stools score) / 3 [26]

The scores were given according to S2 Table.

### 2.5 Colonic hematoxylin-eosin (H&E) staining, alien-blue and periodic acid-Schiff (AB-PAS) staining, and Masson’s trichrome (Masson) staining

The colonic tissues of the mice were collected and fixed in 4% paraformaldehyde, embedded in paraffin, and cut into 4 μm thick sections. Treated the sections with xylene I for 15 min and xylene II for 15 min; followed by 100% ethanol I, 100% ethanol II, 95% ethanol I, 95% ethanol II, and 75% ethanol for 2 min each. According to the reagent instructions, H&E staining for histopathological examination of colonic inflammation, AB-PAS staining for colonic goblet cells, and Masson staining for collagen fibers in the tissue. After sealing the slices with neutral gum, images were observed and captured using a slide scanner (Olympus SLIDEVIEW VS200, Japan).

### 2.6 Real-time quantitative PCR (RT-qPCR)

Genomic RNA was extracted from mouse colon tissues and small intestinal organoid tissues using the Trizol protocol. The concentration of RNA was detected using a NanoDrop 2000 UV-vis spectrophotometer (Thermo Fisher Scientific, Waltham, MA, USA). Subsequently, the genomic RNA was reversely transcribed into cDNA, and the amplification was detected using the SYBR RT-qPCR Kit in a real-time fluorescence quantitative PCR instrument (CFX96, Bio-Rad, Hercules, CA, USA). The amplification protocol consisted of an initial denaturation step at 95°C for 5 min, followed by 39 cycles of denaturation at 95°C for 10 s, and annealing at 60°C for 30 s. The relative mRNA transcription levels of *IL-6*, *IL-1β*, *TNF-α*, *IFN-γ*, *IL-10*, and *Lgr5* were calculated by the 2^-ΔΔCt^ method, and *GAPDH* was used as the internal control for normalization. The primer sequences are listed in S1 Table.

### 2.7 Immunohistochemistry (IHC) and immunofluorescence (IF)

The paraffin-embedded sections of mouse colonic tissues were first deparaffinized using xylene and graded ethanol. Antigen retrieval was then performed by boiling the sections in 1x EDTA antigen retrieval solution for 20 min. Following this, the sections were treated with 3% endogenous peroxidase enzyme for 10 min to block endogenous peroxidase activity. For IHC, the sections were blocked with 5% goat serum for 30 min before incubation overnight with primary antibodies specific for MUC2 (1:2,000), Occludin (1:200), ZO-1 (1:2,000), and PCNA (1:200). On the next day, the sections were incubated with the appropriate secondary antibody (1:500), followed by DAB staining. Finally, the sections were mounted with neutral gum and observed using a slide scanner (Olympus SLIDEVIEW VS200, Japan). For IF, the sections were permeabilized with 0.3% Triton X-100 for 30 min and blocked with 5% BSA for 1 h. They were then incubated overnight with primary antibodies respectively specific for Lgr5 (1:100), PCNA (1:200), MUC2 (1:500), Dclk1 (1:200), and ChgA (1:500). On the next day, the sections were incubated with the fluorescent secondary antibody 488 (1:200) and mounted by DAPI solution. Finally, they were mounted with an anti-fade mounting medium and observed using an upright fluorescence microscope (Eclipse 80i, Nikon Ltd, Japan).

### 2.8 Western blotting (WB)

The mouse colonic tissues and small intestinal organoid tissues were digested with RIPA lysis buffer. After measuring the concentration, an equal amount of protein samples was loaded and isolated using SDS-PAGE. Next, transfer the proteins onto a PVDF membrane. After blocking with 5% non-fat milk, separately incubated the membrane with the primary antibodies Occludin (1:10,000), ZO-1 (1:20,000), CYP1A1 (1:1,000), IL-22 (1:500), Lgr5 (1:1,000), and PCNA (1:2,000) at 4°C overnight. Then incubated with the secondary antibody (1:10,000) for 1 h at room temperature. Finally, Super ECL Detection Reagent was utilized for sensitive detection of the protein.

### 2.9 Statistical analysis

Semi-quantitative statistics used Image J software (version 1.53i, US National Institutes of Health, USA) after image acquisition. The statistical analysis and graphical representation were performed using GraphPad Prism 8.0. The data were presented as mean + SD. Measurements were first subjected to normality tests, and the Homogeneity of Variance Test was performed between groups. Variance irregularities using the Mann-Whitney U-test. One-way analysis of variance (ANOVA) was used to test differences between multiple groups, Mann-Whitney U-test and t-test were used to compare differences between two groups. The *p* value less than 0.05 means a statistically significant difference; ns means no statistically significant.

## 3. Results

### 3.1. Preliminary identification of HnAg and experimental design

**Fig 1:**
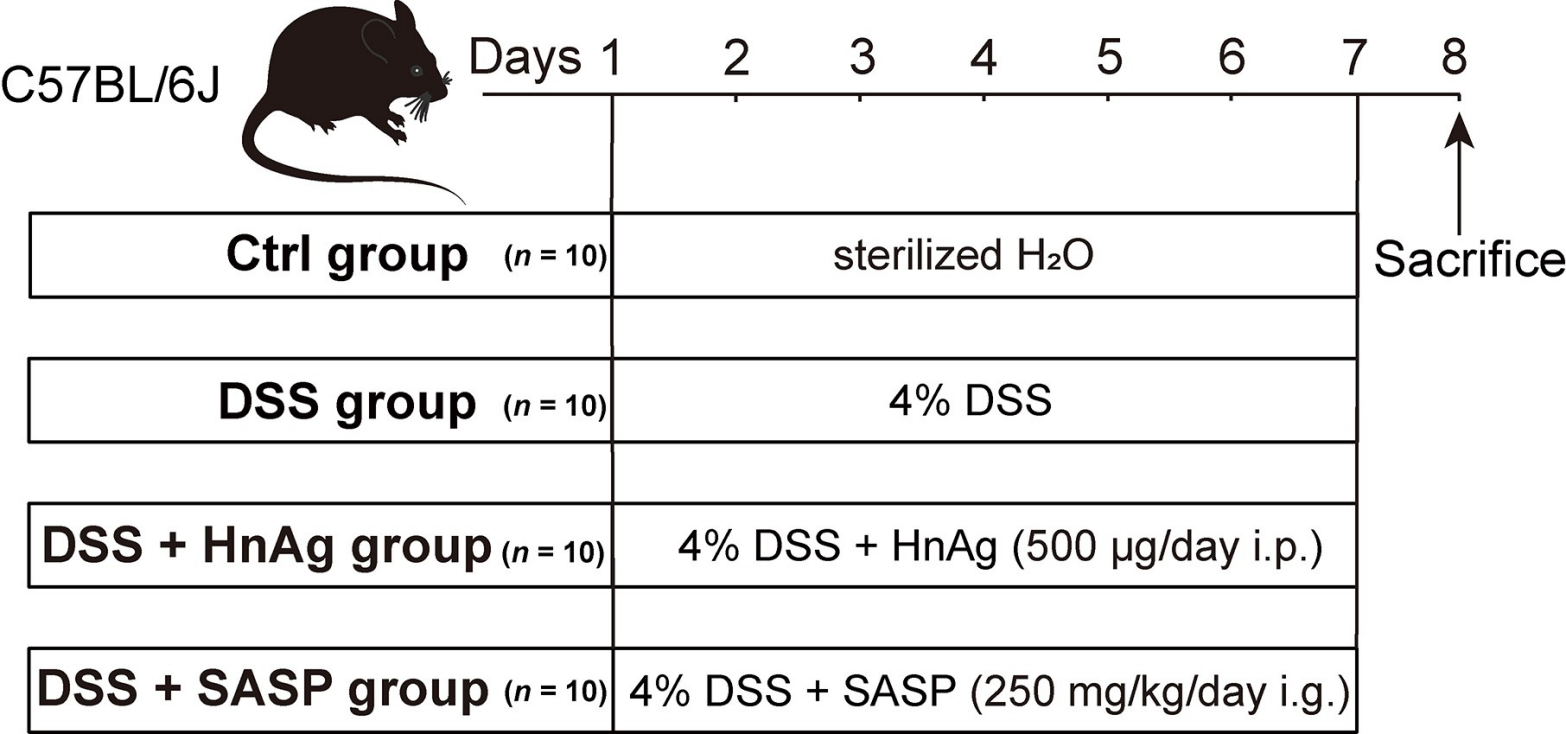
Experimental flowchart. The Ctrl group mice were given sterilized H_2_O and the DSS group mice were given 4% DSS in sterilized H_2_O (*ad libitum*) for 7 days; The DSS + HnAg group or the DSS + SASP group mice were given 4% DSS in sterilized H_2_O (*ad libitum*) along with intraperitoneal injections of 500 μg/day of HnAg or 250 mg/kg/day of SASP via oral gavage for 7 days. All mice were anesthetized and sacrificed on the 8^th^ day.

Eggs were round or suborbicular, with a thin shell containing a thicker germinal membrane, inside which small hooks could be seen; Suckers, parietal protrusions, and small hooks were seen in the cephalic segment of the parasites; the proglottids contained ovaries and testes; the gravid proglottids were filled with eggs. The products of PCR amplification of the *H*. *nana COX-I* gene were subjected to agarose gel electrophoresis, a clear band was seen at 202 bp, and the parasites were identified as *H*. *nana* (S1B–S1F Fig). The results revealed that there were many proteins in the HnAg, but four major bands with relatively high expression levels were observed, located at 25-35kDa, 35-45kDa, 72-100kDa, and 100-140kDa, with the highest expression observed between 100kDa-140kDa (S1G Fig). To study the effect of HnAg on UC, this experiment was carried out in mice using the experimental method shown in (Fig 1).

### 3.2. HnAg alleviates clinical signs in UC mice

**Fig 2:**
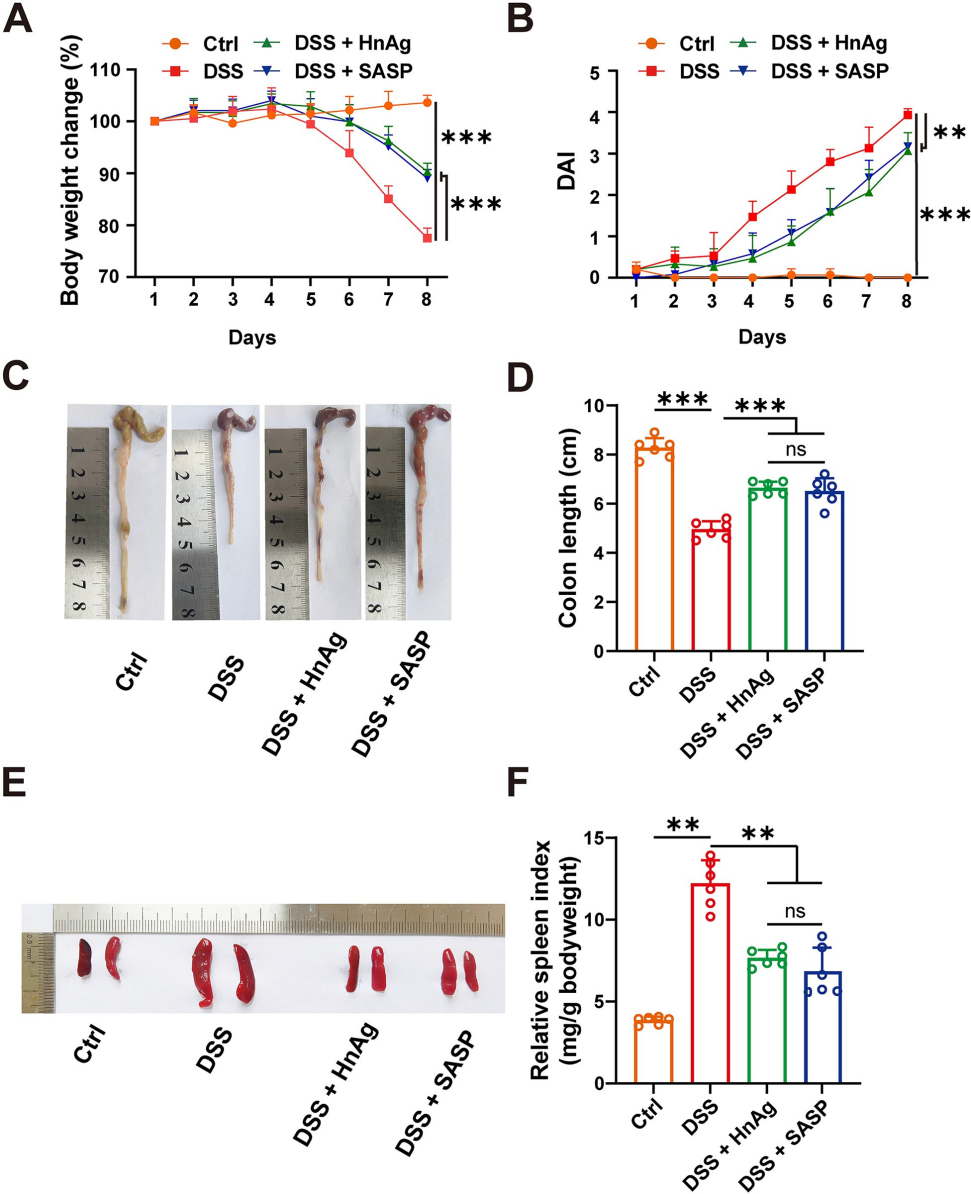
Effects of HnAg on clinical symptoms of UC mice. (**A**) Changes in mouse body weight (%); (**B**) DAI score; (**C**) Representative colon images; (**D**) Colon length (cm); (**E**) Representative spleen images; (**F**) Spleen index. Data are presented as mean + SD in (A), (B), (D), and (F), *n* = 10 per group for (A) and (B), *n* = 6 per group for (D) and (F), ** *p* < 0.01, *** *p* < 0.001, ns: not statistically significant.

Weight loss, colon shortening, diarrhea, and intestinal bleeding are typical clinical symptoms of UC. We evaluated these changes to determine the progression of UC in mice, compared to the Ctrl group, the DSS group had greater weight change, higher DAI scores, and intestinal bleeding. The results showed that the colitis model was successfully established (S1H–S1J Fig). Compared with the Ctrl group, mice in the DSS group exhibited significantly reduced body weight and increased DAI scores (Fig 2A and 2B). The colon lengths of mice in the DSS group were significantly shortened, and splenomegaly was observed (Fig 2C and 2D). After intervention with HnAg or SASP, the body weight of mice was significantly higher than the DSS group, and the DAI score and spleen index were significantly reduced (Fig 2E and 2F). Additionally, HnAg or SASP treatment reduced DSS-induced colon shortening. These results indicate that intervention with HnAg or SASP can alleviate clinical symptoms in UC mice.

### 3.3. HnAg improves histopathological changes in UC mice

**Fig 3:**
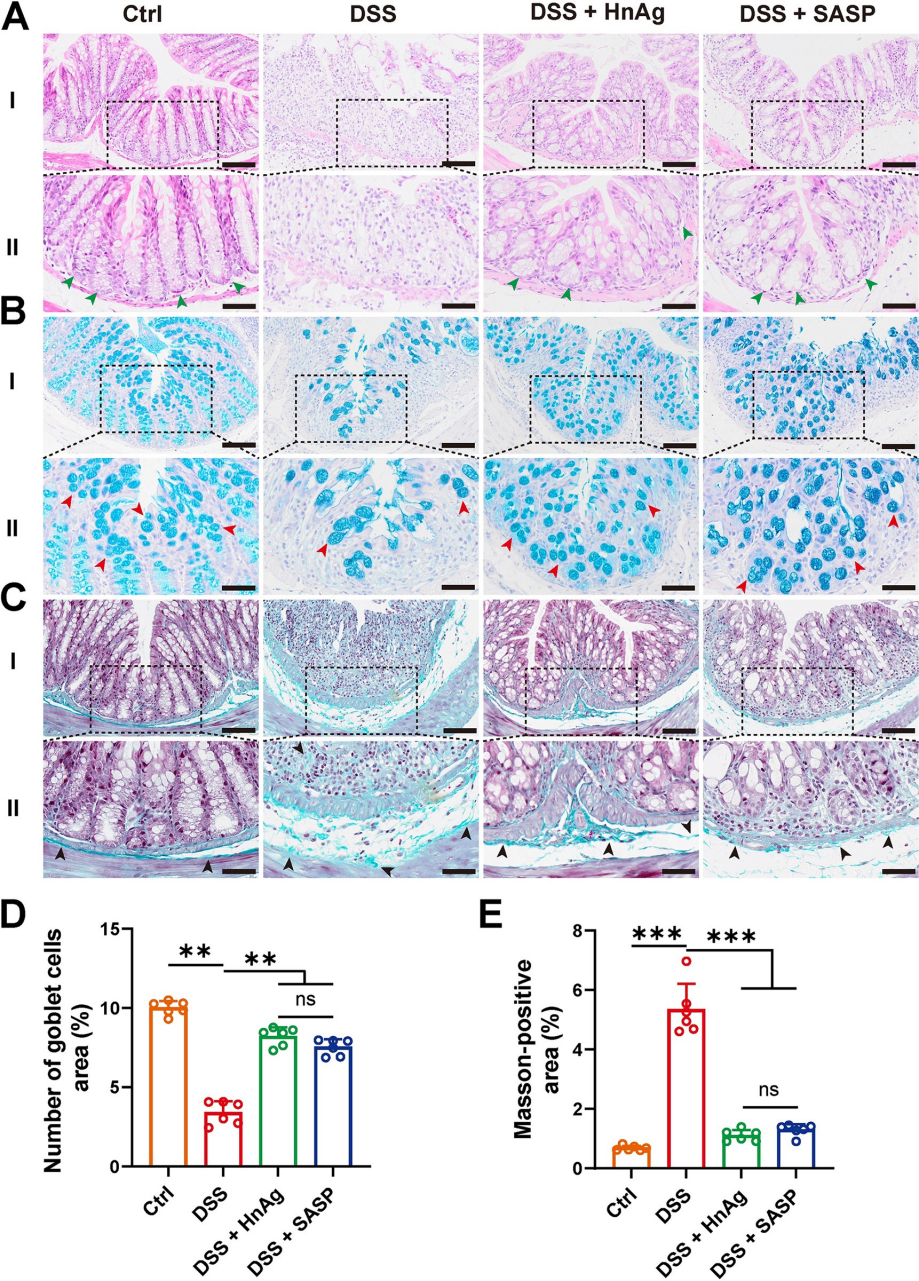
HnAg improves the histopathological changes of UC mice. (**A**) Representative images of H&E-stained. The crypts are indicated by green arrows; (**B**) Representative images of AB-PAS-stained. The goblet cells are indicated by red arrows; (**C**) Representative images of Masson-stained. The collagen fibers are indicated by black arrows. Colon tissues of (A), (B), and (C) were from Ctrl, DSS, DSS + HnAg, and DSS + SASP groups respectively (scale bars 100 μm for I, and 50 μm for II); (**D**) Number of goblet cells in colonic tissue; (**E**) Percentage of Masson-positive area was semi-quantified using Image J software. Data are presented as mean + SD for (D) and (E), *n* = 6 per group, ** *p* < 0.01, *** *p* < 0.001, ns: not statistically significant.

Histopathological changes in mouse colonic tissues were observed using H&E staining, AB-PAS staining, and Masson staining. The results of H&E staining showed that the mice in the DSS group had obvious colonic tissue damage, mucosal ulceration, inflammatory cell infiltration, and the integrity of the crypts was damaged, the colonic tissue damage of the mice was significantly improved after intervention with HnAg or SASP (Fig 3A). Compared with the Ctrl group, the DSS group showed a significant reduction in goblet cells, and Masson staining revealed a significant increase in fibrosis. However, the number of goblet cells in the DSS + HnAg group and DSS + SASP group showed partial recovery, and the degree of fibrosis was significantly reduced (Fig 3B–3E). These results indicate that intervention with HnAg or SASP alleviates histopathological damage in DSS-induced mice. Compared with SASP, pathological damage and goblet cell counts in the intestines of mice appeared to recover better after HnAg intervention, although not statistically significant.

### 3.4. Effects of HnAg on the intestinal mucosal barrier in UC mice

**Fig 4:**
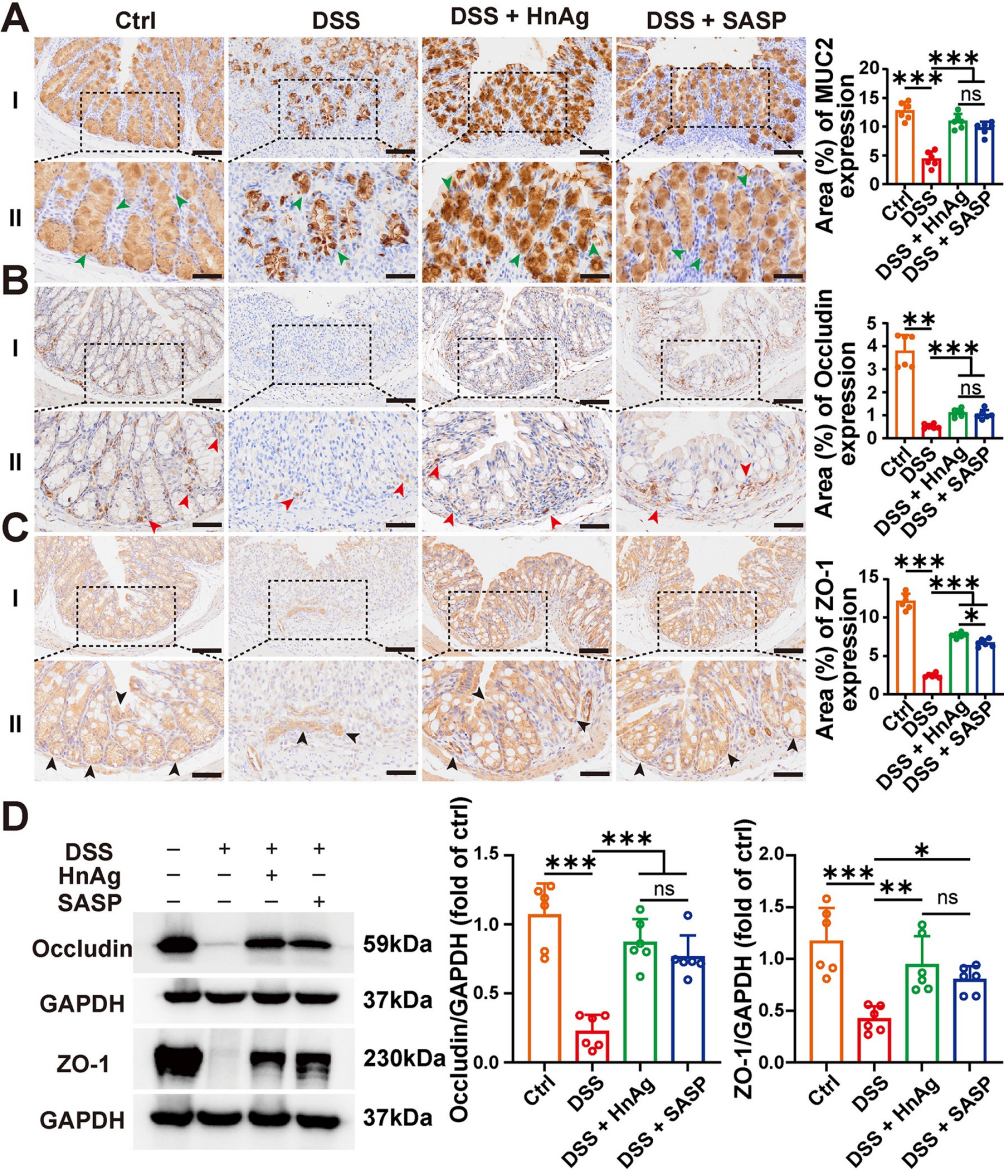
HnAg improves gut barrier integrity. Representative images of IHC with MUC2 **(A)**, Occludin **(B)**, and ZO-1 **(C)**; Percentages of MUC2, Occludin, and ZO-1-positive areas were semi-quantified using Image J software; Proteins were stained brown and judged to be positive by green, red, and black arrows, colon tissues were from Ctrl, DSS, DSS + HnAg, and DSS + SASP groups (scale bars 100 μm for I, and 50 μm for II); (**D**) The protein expression level of Occludin and ZO-1 (left panel); Percentage of the relative expression of Occludin and ZO-1 were semi-quantified using Image J software (right panel). Data are presented as mean + SD for the right panel of (A-D), *n* = 6 per group for the right panel of (A-D), * *p* < 0.05, ** *p* < 0.01, *** *p* < 0.001, ns: not statistically significant.

Regulation of tight junction proteins is a vital component of epithelial barrier repair after injury. To investigate whether HnAg could improve intestinal barrier function, the alterations of mucins and tight junction proteins in the colon tissues of each group were analyzed by IHC. Additionally, WB was used to further verify changes in tight junction proteins. The mucin MUC2 secreted by goblet cells plays a significant role in maintaining intestinal homeostasis. The results showed a significant reduction in mucin in the DSS group, possibly due to the depletion of goblet cells in UC, leading to insufficient secretion of mucin. Intervention with HnAg and SASP increased mucin secretion (Fig 4A). DSS-induced damage to the intestinal mucosal barrier is closely related to the disorganization of colonic tight junction proteins, such as ZO-1 and Occludin [27]. It was found that the expression of Occludin and ZO-1 proteins was abnormally reduced in the DSS group, while the expression of tight junction proteins was significantly increased in the DSS + HnAg group and DSS + SASP group (Fig 4B and 4C). WB results also showed severe damage to tight junction proteins in the DSS group, which was increased after intervention with HnAg or SASP (Fig 4D). These results indicate that HnAg can improve intestinal barrier dysfunction induced by DSS by protecting mucin and tight junction proteins, thereby restoring the integrity of the epithelial structure.

### 3.5. Effects of HnAg on intestinal inflammation in UC mice

**Fig 5:**
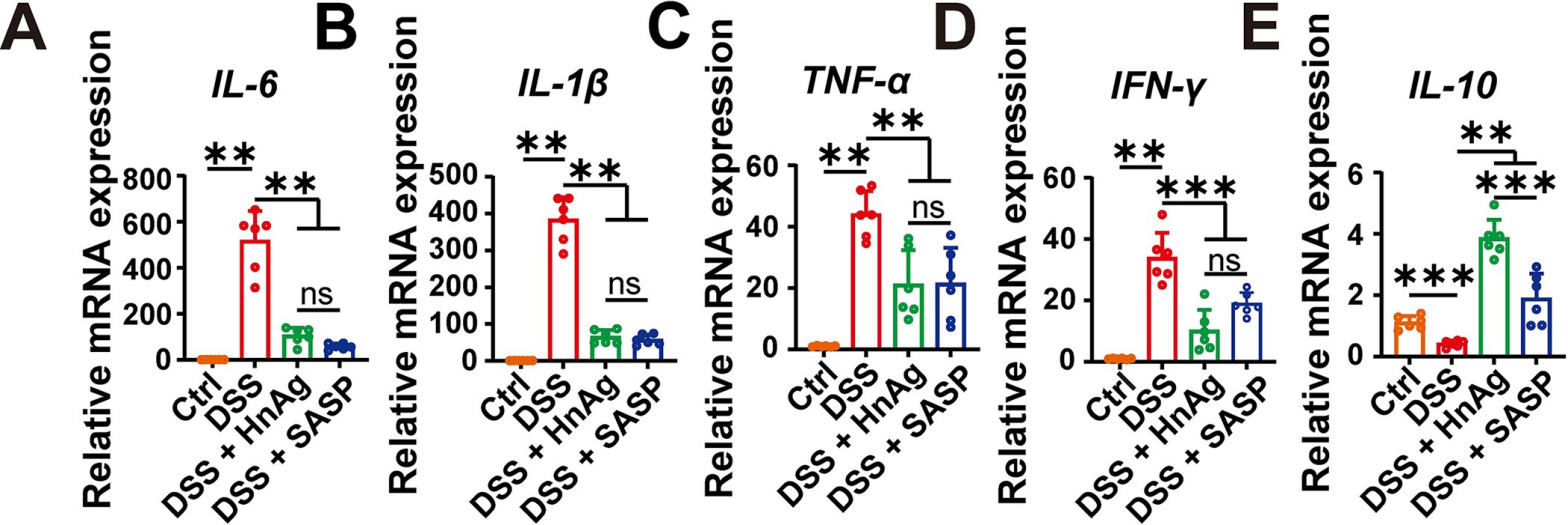
HnAg protects UC mice by inhibiting intestinal inflammation. The transcription levels of *IL-6* (**A**); *IL-1β* (**B**); *TNF-α* (**C**); *IFN-γ* (**D**); and *IL-10* (**E**). The relative quantification was determined using the 2^-ΔΔCt^ method normalized to *GAPDH*. Data are presented as mean + SD for (A)-(E), *n* = 6 per group for (A)-(E), ** *p* < 0.01, *** *p* < 0.001, ns: not statistically significant.

Pro-inflammatory cytokines and anti-inflammatory cytokines play key roles in the pathogenesis of UC, and overproduction of pro-inflammatory cytokines is a hallmark of UC colon injury, IL-10 is an anti-inflammatory cytokine, and an exacerbation of the inflammatory response will restrict its production. Compared with the Ctrl group, the gene transcription levels of pro-inflammatory cytokines *IL-6*, *IL-1β*, *TNF-α*, and *IFN-γ* were significantly upregulated in the DSS group (Fig 5A–5D), while the anti-inflammatory cytokine *IL-10* was decreased (Fig 5E). In contrast, HnAg or SASP intervention inhibited the upregulation of pro-inflammatory cytokine expression levels in colonic tissues induced by DSS while promoting the up-regulation of anti-inflammatory factor *IL-10* expression levels.

### 3.6 HnAg activates the AhR/IL-22 signaling pathway to promote ISCs proliferation

**Fig 6:**
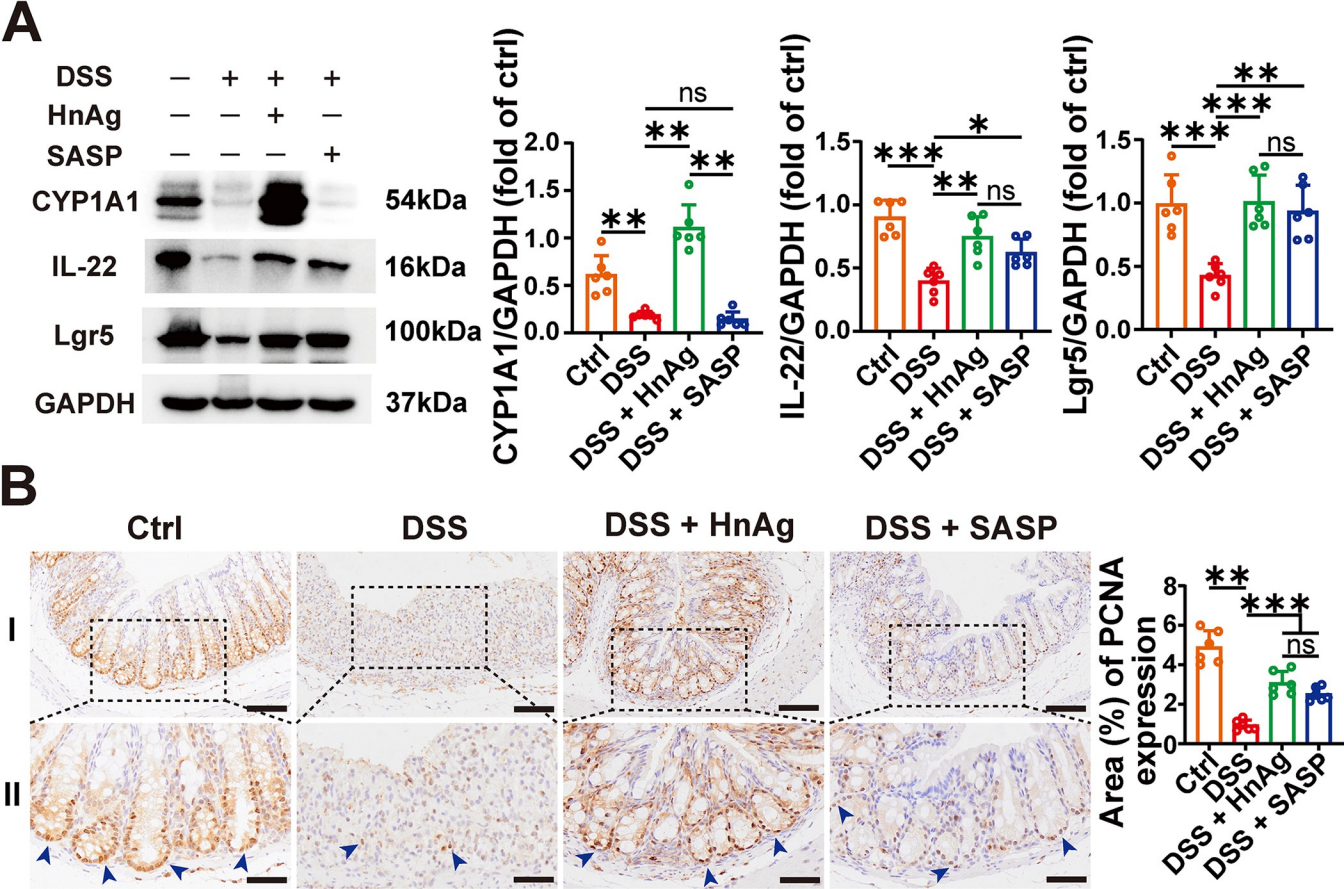
HnAg promotes ISCs proliferation by activating the AhR/IL-22 signaling pathway. (**A**) The protein expression level of CYP1A1, IL-22, and Lgr5 (left panel); Percentage of the relative expression of CYP1A1, IL-22, and Lgr5 were semi-quantified using Image J software (right panel). (**B**) Representative images of IHC with PCNA and percentage of PCNA-positive area were semi-quantified using Image J software (scale bars 100 μm for I, and 50 μm for II); Proteins were stained brown and judged to be positive by blue arrows. Data are presented as mean + SD at the right panel of (A-B), *n* = 6 per group for the right panel of (A-B), * *p* < 0.05, ** *p* < 0.01, *** *p* < 0.001, ns: not statistically significant.

CYP1A1 is a downstream target of AhR, and the activation status of AhR can be assessed by detecting expression changes of CYP1A1. Previous studies have shown that AhR can activate downstream IL-22, which acts on tight junction proteins and ISCs to protect the intestinal mucosal barrier [8, 9]. Compared with the Ctrl group, the protein expression levels of CYP1A1, IL-22, and Lgr5 were decreased in the DSS group. However, after intervention with HnAg, the expression levels of these proteins were increased. SASP intervention increased the protein expression levels of IL-22 and Lgr5 but did not alter the expression of CYP1A1 protein (Fig 6A). Additionally, we also found that DSS caused a sharp decrease in the number of PCNA cells in mice, while HnAg and SASP intervention could increase the number of PCNA cells (Fig 6B), suggesting that HnAg improved the proliferation of intestinal epithelium. In summary, HnAg promotes the increase of ISCs by activating the AhR/IL-22 signaling pathway.

### 3.7. HnAg promotes increased differentiation of ISCs to alleviate UC in mice

**Fig 7:**
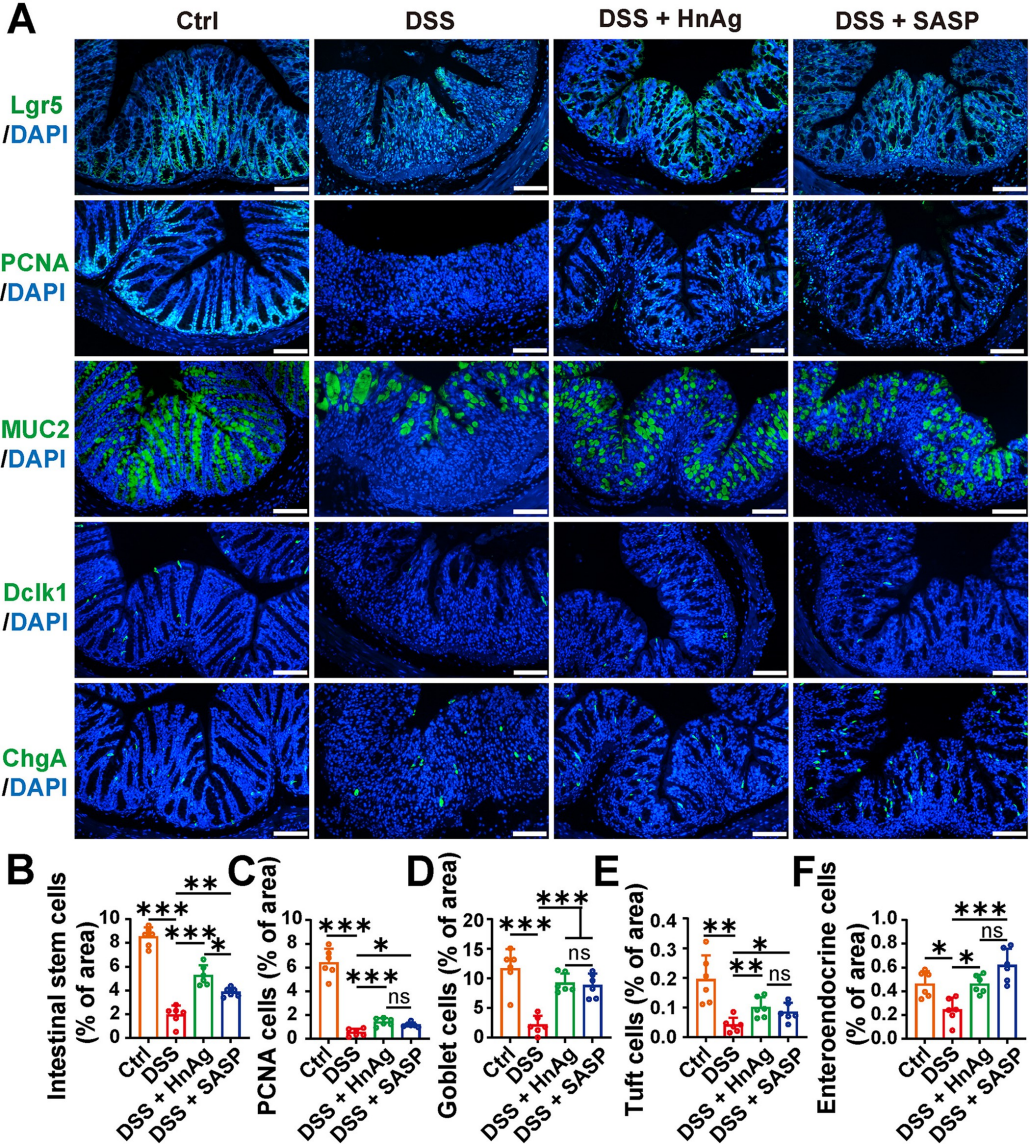
HnAg alleviates UC in mice by promoting increased differentiation of ISCs. (**A**) Representative images of IF with Lgr5, PCNA, MUC2, Dclk1, and ChgA (scale bars 100 μm); The green color represents the Lgr5, PCNA, MUC2, Dclk1, and ChgA respectively, and the blue color represents the nucleus. Percentages of Lgr5 (**B**), PCNA (**C**), MUC2 (**D**), Dclk1 (**E**), and ChgA (**F**) -positive areas were semi-quantified using Image J software. Data are presented as mean + SD for (B)-(F), *n* = 6 per group for (B)-(F), * *p* < 0.05, ** *p* < 0.01, *** *p* < 0.001, ns: not statistically significant.

The renewal and differentiation of ISCs play a key role in improving the repair and regeneration of the intestinal epithelium. ISCs located in the intestinal crypts generate transit-amplifying (TA) cells, which differentiate into all types of intestinal epithelial cells [28]. Previously, we found that HnAg could activate the AhR/IL-22 signaling pathway to alter the protein Lgr5. To further elucidate whether HnAg affects the renewal and differentiation of ISCs, we used IF to observe ISCs markers (Lgr5), proliferation cell markers (PCNA), and the expression of different intestinal epithelial cell markers, including goblet cells (MUC2), tuft cells (Dclk1), and enteroendocrine cells (ChgA). The results revealed that the number of ISCs and proliferating cells decreased in the DSS group, along with a decrease in the number of goblet cells, tuft cells, and enteroendocrine cells. However, after intervention with HnAg or SASP, the number of the above cells was restored, and the number of ISCs recovered more after HnAg intervention (Fig 7). These findings confirm that HnAg can regulate the renewal and differentiation of ISCs in UC mice.

### 3.8. HnAg attenuates DSS-induced small intestinal organoid damage

**Fig 8:**
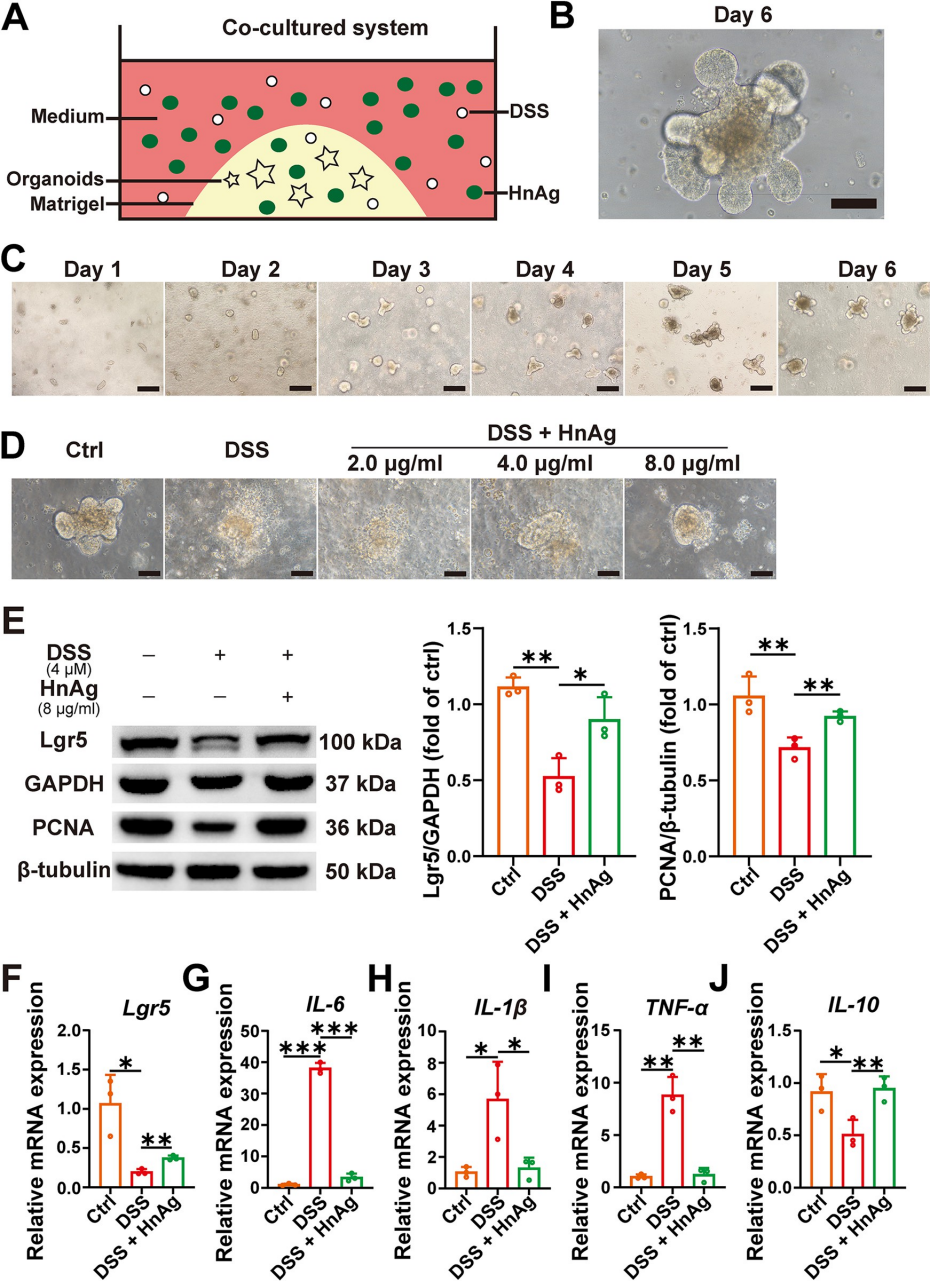
HnAg attenuates DSS-induced small intestinal organoid damage by increasing ISCs and inhibiting inflammatory responses. (**A**) Schematic diagram of in vitro culture of mouse small intestine-like organoids. The mouse small intestinal crypts were extracted, and the crypt suspension was mixed with an equal volume of Matrigel, and then seeded into 24-well culture plates according to a suspension of 30 μl per well, with the addition of complete medium for small intestinal organoids, and the fluid was changed every two days. After 3 days of culture of the small intestinal organoids, 4 μM DSS was added and cultured for 24 h. Then, different doses of HnAg were added to continue the co-culture for 24 h. (**B**) Successfully cultured mouse small intestine-like organoids were observed under the microscope on the 6^th^ day (scale bar 100 μm). (**C**) The growth status of organoids was observed by optical microscope at 1–6 days (scale bars 200 μm). (**D**) The morphology of the organoids on the 6^th^ day (scale bars 100 μm) in the Ctrl, DSS, DSS + HnAg (2 μg/ml, 4 μg/ml, and 8 μg/ml) groups, which were treated with DSS or HnAg. (**E**) The protein expression levels of Lgr5 and PCNA (left panel); Percentage of the relative expression of Lgr5 and PCNA were semi-quantified using Image J software (right panel). The transcription levels of *Lgr5* (**F**), *IL-6* (**G**), *IL-1β* (**H**), *TNF-α* (**I**), and *IL-10* (**J**) were determined using the 2^-ΔΔCt^ method normalized to *GAPDH*. Data are presented as mean + SD for the right panel of (E) and (F)-(J), *n* = 3 per group, * *p* < 0.05, ** *p* < 0.01, *** *p* < 0.001.

The role of HnAg in attenuating inflammation in vitro was investigated in a DSS-induced inflammation model of small intestinal-like organoids in mice, as shown in Fig 8A. The morphology of the organoids was daily observed under the microscope, and the organoids gradually grew from crypts to “crypt-villus” structures, and successfully cultured mouse small intestinal organoids exhibited intricate but natural budding structures were presented (Fig 8B and 8C). DSS caused damage to small intestinal organoid structures, and the organoids did not maintain their typical morphologic structure, whereas the intervention of HnAg dose-dependently attenuated DSS-induced damage, and at 8 μg/ml, the organoids maintained their normal morphologic structure and the emergent structures could be visualized (Fig 8D). Therefore, we chose this dose for the next study, and by WB detection of ISCs marker (Lgr5) and proliferating cell marker (PCNA), we found HnAg could restore the reduction of ISCs caused by DSS, which was also confirmed by the results of RT-qPCR (Fig 8E and 8F). In addition, we also found that HnAg could decrease the expression of pro-inflammatory factors (*IL-6*, *IL-1β*, and *TNF-α*) while increasing the expression of anti-inflammatory factor *IL-10*. Such results highly validated that HnAg attenuated DSS-induced damage to small intestinal organoids in mice, mainly by restoring DSS-induced reduction in ISCs and attenuating the inflammatory response.

## 4. Discussion

UC is an inflammatory bowel disease of unknown etiology, and the incidence of UC varies greatly in different regions and is increasing globally [29]. The most common symptoms of UC are bloody diarrhea, often accompanied by abdominal pain and urgency [30]. Helminth infections can induce immune responses in mammalian hosts, usually modulating this response to promote parasite survival and inhibit expulsion [31]. While helminth-induced immune modulation is beneficial for the parasites, it can also have favorable effects on host immune function [32]. Helminths can exploit the innate immune system to modulate colitis [33]. Extracts and associated products of helminths’ tissues can ameliorate the severity of inflammatory diseases in mouse model systems [19, 34, 35]. The excretory-secretory products of *Trichinella spiralis* and the crude extract antigen of *H*. *diminuta* have been shown to alleviate UC [19, 36]. In this study, We first identified *H*. *nana* by morphology and the mitochondrial cytochrome c oxidase I (*COX-I*) gene of *H*. *nana*, which is an important mitochondrial gene for parasites with high sequence variability and specificity among different parasites [37]; *COX-I* is the slower evolutionary rate in the mitochondrial genome, and it has been applied by many scholars as a molecular marker for taxonomic identification of parasites [38]. In addition, the moderate sequence length of the *COX-I* gene and the high feasibility of PCR amplification and sequencing techniques also facilitate its application in the molecular identification of parasites.

In addition, we assessed the quantity and molecular size of the proteins contained in HnAg by immunoblotting. Then, we evaluated the therapeutic effects of HnAg on UC using a DSS-induced UC mouse model. SASP, an effective drug for the treatment of UC, was used as a positive control [20]. We found that both HnAg and SASP intervention significantly reduced corresponding clinical symptoms of UC and pathological damage. Compared with SASP, although the overall effect of HnAg in alleviating UC in mice was about the same, it appeared to be more favorable in some respects. Particularly in terms of protecting the intestinal mucosa and restoring the number of ISCs. In addition, we used a dose of 500 μg of HnAg much lower than the 250 mg/kg of SASP. More importantly, HnAg is a newly discovered substance that can alleviate UC, which has not been studied before. We demonstrated that HnAg enhanced the expression of intestinal mucins and tight junction proteins, thereby maintaining the integrity of the intestinal mucosal barrier. It also promoted ISCs proliferation and differentiation by regulating the AhR/IL-22 signaling pathway, thereby accelerating epithelial cell regeneration and repair.

The pathogenesis of UC involves multiple processes, including genetic susceptibility, mucosal immune dysregulation, epithelial barrier dysfunction, disruption of intestinal microbiota, and environmental factors [39]. Although the etiology of UC is not clear, the ultimate symptom is the destruction of the intestinal barrier. Therefore, restoring intestinal barrier function is the primary therapeutic goal, which can be achieved by promoting inflammation resolution and accelerating mucosal repair [40]. The colonic barrier is mainly composed of tight junction proteins, mucus, antimicrobial peptides, and immune cells or molecules [41, 42]. The decreased function of the intestinal mucosal barrier and the disruption of tight junction protein integrity are the major pathological bases of UC. Tight junction proteins, a membrane protein complex composed of Occludin, Claudins, and ZOs, connect adjacent cells to form the epithelial barrier [43]. MUC2 is a secretory mucin produced by goblet cells and is a major component of the mucus layer [44]. The mucus layer serves as the first line of defense in the intestinal barrier and plays a key role in intestinal defense [45]. Our results indicated that HnAg intervention could alleviate the clinical symptoms and pathological damage in UC mice. Additionally, HnAg intervention could reduce goblet cell loss, promote colonic mucin secretion, and increase the expression of the major mucin MUC2. Furthermore, the tight junction proteins Occludin and ZO-1 were significantly decreased in the UC model, while HnAg could increase the expression of tight junction proteins. Therefore, it was inferred that HnAg improved UC by maintaining the integrity of the intestinal barrier.

Mucosal healing in UC is closely related to the repair capacity of the intestinal epithelium, which is generated by the renewal and differentiation of ISCs [46]. Aloe vera gel increases the number of ISCs and promotes their differentiation into intestinal epithelial cells, goblet cells, and enteroendocrine cells, thus repairing damaged intestinal epithelium and alleviating DSS-induced colitis in mice [47]. Cells derived from mesenchymal stem cells can significantly promote the regeneration and proliferation of ISCs, thereby reshaping the intestinal epithelial structure of DSS-induced colitis mice [48]. In this study, our data supported that HnAg intervention significantly upregulated the protein expression of the ISCs molecular marker Lgr5 in UC mice. Measurement of the PCNA-positive area to assess epithelial proliferation capacity revealed that HnAg increased the expression of PCNA in UC mice. This evidence confirmed that HnAg could enhance the intestinal mucosal barrier function in UC mice by regulating the proliferation of ISCs.

AhR is a heterodimeric transcription factor widely present in vertebrates, belonging to the basic helix-loop-helix (BHLH) superfamily. It is a potential target for mediating intestinal immune diseases and has been demonstrated to have a protective effect on UC [49]. The direct downstream target of AhR is CYP1A1, and changes in CYP1A1 expression levels usually reflect the activation level of AhR. The CYP1A1 expression level was detected by WB, and it was found that HnAg intervention increased the DSS-induced reduction of CYP1A1, whereas there was no significant change with SASP intervention. The upregulation of CYP1A1 thus reflected the activating effect of HnAg on AhR.

By activating AhR, it in turn regulates the expression of its downstream signaling molecule IL-22, the homeostasis of ISCs and barrier integrity is maintained, inflammation damage is relieved, and intestinal environment balance is regulated [50]. In the DSS-induced colitis animal model, it was found that mice lacking the AhR gene were more susceptible to colitis than wild-type mice [51]. Our research results suggested that the expression level of IL-22 decreased in colitis, while after HnAg intervention, the IL-22 protein expression level increased. We also found that HnAg could activate AhR, suggesting that HnAg could activate the AhR/IL-22 signaling pathway.

To further validate whether HnAg can promote the differentiation of ISCs through the AhR/IL-22 signaling pathway, changes in different cells were verified through IF. The results indicated that DSS damaged ISCs and proliferation cells, leading to a decrease in the number of intestinal secretory cell lineages, such as goblet cells, tuft cells, and enteroendocrine cells. After intervention with HnAg, the number of these cells was partially restored, indicating that HnAg could induce differentiation of ISCs into various intestinal epithelial cells in response to intestinal injury.

Organoids can mimic organ development and fully demonstrate the self-renewing properties of stem cells [52]. Intestinal organoids are consistent with the crypt-villus structure and can reproduce various types of intestinal epithelium cells [53]. Therefore, intestinal organoids provide a good tool for studying the mechanisms related to ISCs and intestinal epithelial repair. In this study, we showed that HnAg could attenuate DSS-induced damage to small intestinal organoids in mice, increase the number of ISCs and proliferating cells, and simultaneously decrease the expression of pro-inflammatory factors and increase the expression of anti-inflammatory factors. The results of in vitro organoid experiments suggest that HnAg can repair the damaged intestinal epithelium via ISCs to alleviate inflammation.

In conclusion, we demonstrated that intraperitoneal injection of HnAg in mice could alleviate UC by inhibiting inflammation and protecting the intestinal mucosal barrier. In vitro models of organoid inflammation also suggested that HnAg increased ISCs and alleviated inflammatory responses. Furthermore, we found that HnAg could activate the AhR/IL-22 signaling pathway to promote the proliferation and differentiation of ISCs into various intestinal secretory cells. This suggested that HnAg could be a therapeutic option for UC.

### Limitation

Although we briefly described the size of the major proteins inside HnAg, we did not clarify exactly which protein or proteins were playing key roles in HnAg. However, previous studies have also explored the mitigating effects of UC in mice by means of maggot extracts, *H*. *diminuta* crude antigens, and *Trichinella spiralis* crude protein [19,23,54]. Next, we need to further screen the proteins that play key roles to provide a scientific basis for the future use of *H*. *nana*-related products for the treatment of UC.

## 5. Conclusions

**Fig 9:**
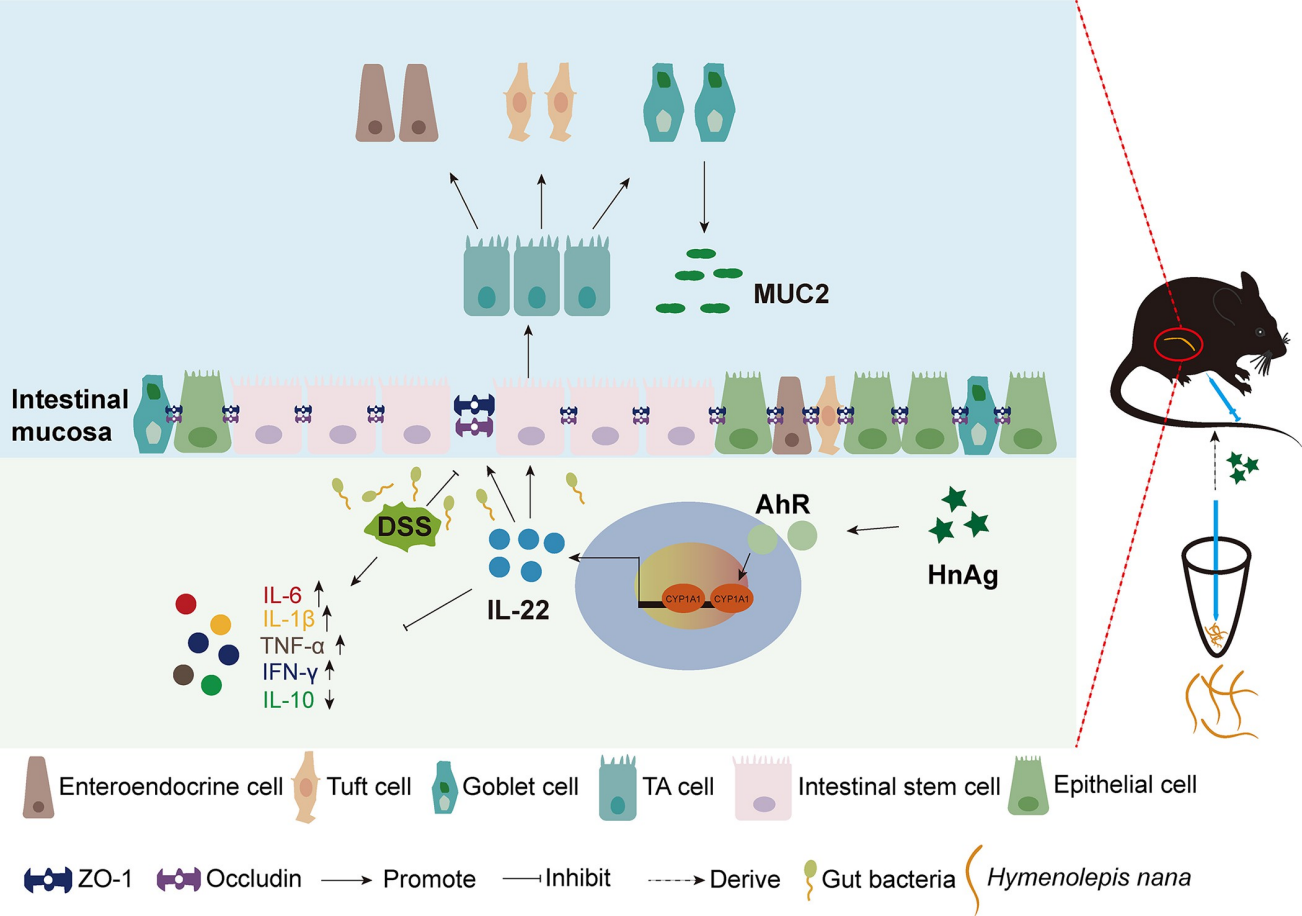
The pathway of action of HnAg in treating UC mice. HnAg was collected after grinding the *H*. *nana* with a handheld homogenizer and injecting them intraperitoneally into mice with UC. HnAg can activate the AhR/IL-22 signaling pathway, which leads to increased differentiation of ISCs and tight junctions in the intestinal tract, and thus alleviates the intestinal damage caused by DSS.

In this study, we found that HnAg could activate the AhR/IL-22 signaling pathway to protect the intestinal barrier and promote the proliferation and differentiation of ISCs to alleviate DSS-induced UC in mice (Fig 9). This study preliminarily revealed the alleviating effect of HnAg on colitis in mice, providing a new avenue for considering worms and their associated products as innovative approaches to maintaining intestinal mucosal integrity for UC treatment in the future.

## Supporting information

S1 Fig Identification of *H*. *nana* and establishment of the model of UC. (**A**) Representative picture of the female hamsters (*Phodopus sungorus*); **(B)** Eggs obtained from *H*. *nana* adults (scale bar 20 μm); (**C**) Head joint of *H*. *nana* after staining with carboxyl borate red, the sucker is indicated by red arrow, and the parietal protrusion is indicated by black arrow (scale bar 100 μm); (**D**) Representative image of mature proglottids of *H*.*nana* (scale bar 100 μm); (**E**) Representative image of gravid proglottids of *H*.*nana* (scale bar 100 μm); (**F**) PCR amplification electrophoresis of the *COX-I* gene of *H*. *nana*, M: DL 2000 marker, Lane 1: *COX-I*, Lane 2: sterilized H_2_O; (**G**) Immunoblotting result of HnAg, M: protein marker (10–245 kDa); Lane 1–5: HnAg; (**H**) Mouse body weight change (%); (**I**) Fecal occult blood test, according to the instructions of the Kit, the corresponding reagent was added dropwise to the mouse feces, and the occult blood condition was determined after 1 min. The darker color of the sample means more bleeding; (**J**) DAI score. Data are presented as mean + SD for (H) and (J), *n* = 10 per group for (H) and (J), ** *p* < 0.01, *** *p* < 0.001 compared with the Ctrl group.
(TIF)

S2 Fig Raw data from WB experiment of Figs 4 and 6 in the results of UC experiments in mice. The original graph of the WB experiment, from left to right are Ctrl, DSS, DSS + HnAg, and DSS + SASP groups.
(TIF)

S3 Fig Raw data of the WB experiment of Fig 8 in the results of the mouse small intestine organoid experiment. The data used for graphing in the manuscript, from left to right the Ctrl, DSS, and DSS + HnAg groups.
(TIF)

S1 Table Primer sequences were used for RT-qPCR experiments of the current study. (DOCX)

S2 Table Scoring system for disease activity index (DAI) in the mice. (DOCX)

S3 Table Data used for graphing in manuscripts. (XLSX)
