# Supplementary figures and images for: Hymenolepis nana antigens alleviate ulcerative colitis by promoting intestinal stem cell proliferation and differentiation via AhR/IL-22 signaling pathway
Source: PLoS Negl Trop Dis. 2024 Dec 12;18(12):e0012714. doi: 10.1371/journal.pntd.0012714 (PMC11670978; doi:10.1371/journal.pntd.0012714)

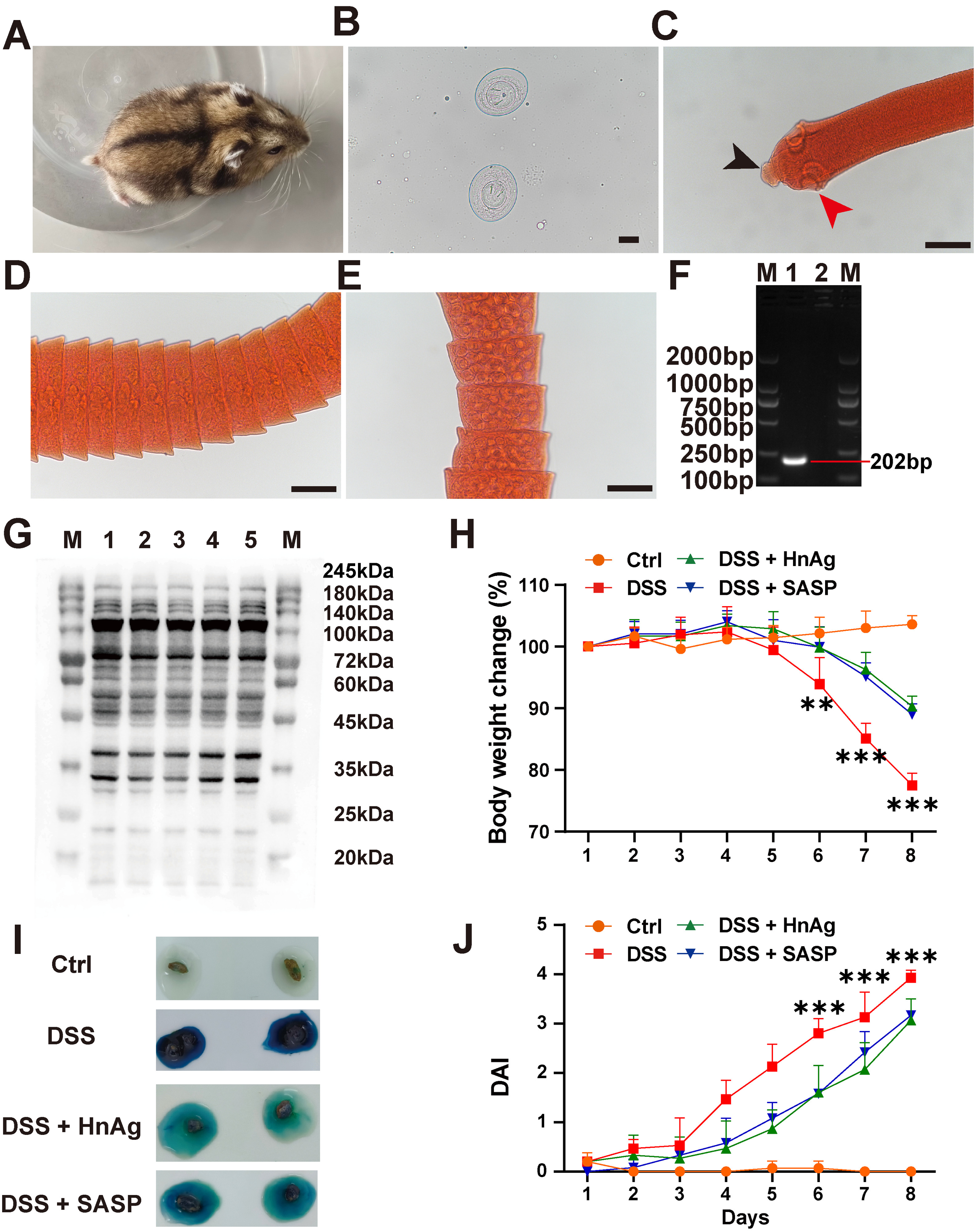

Supplement: S1 Fig — (A) Representative picture of the female hamsters (Phodopus sungorus); (B) Eggs obtained from H. nana adults (scale bar 20 μm); (C) Head joint of H. nana after staining with carboxyl borate red, the sucker is indicated by red arrow, and the parietal protrusion is indicated by black arrow (scale bar 100 μm); (D) Representative image of mature proglottids of H.nana (scale bar 100 μm); (E) Representative image of gravid proglottids of H.nana (scale bar 100 μm); (F) PCR amplification electrophoresis of the COX-I gene of H. nana, M: DL 2000 marker, Lane 1: COX-I, Lane 2: sterilized H2O; (G) Immunoblotting result of HnAg, M: protein marker (10–245 kDa); Lane 1–5: HnAg; (H) Mouse body weight change (%); (I) Fecal occult blood test, according to the instructions of the Kit, the corresponding reagent was added dropwise to the mouse feces, and the occult blood condition was determined after 1 min. The darker color of the sample means more bleeding; (J) DAI score. Data are presented as mean + SD for (H) and (J), n = 10 per group for (H) and (J), ** p < 0.01, *** p < 0.001 compared with the Ctrl group. (TIF) [file pntd.0012714.s001.tif]

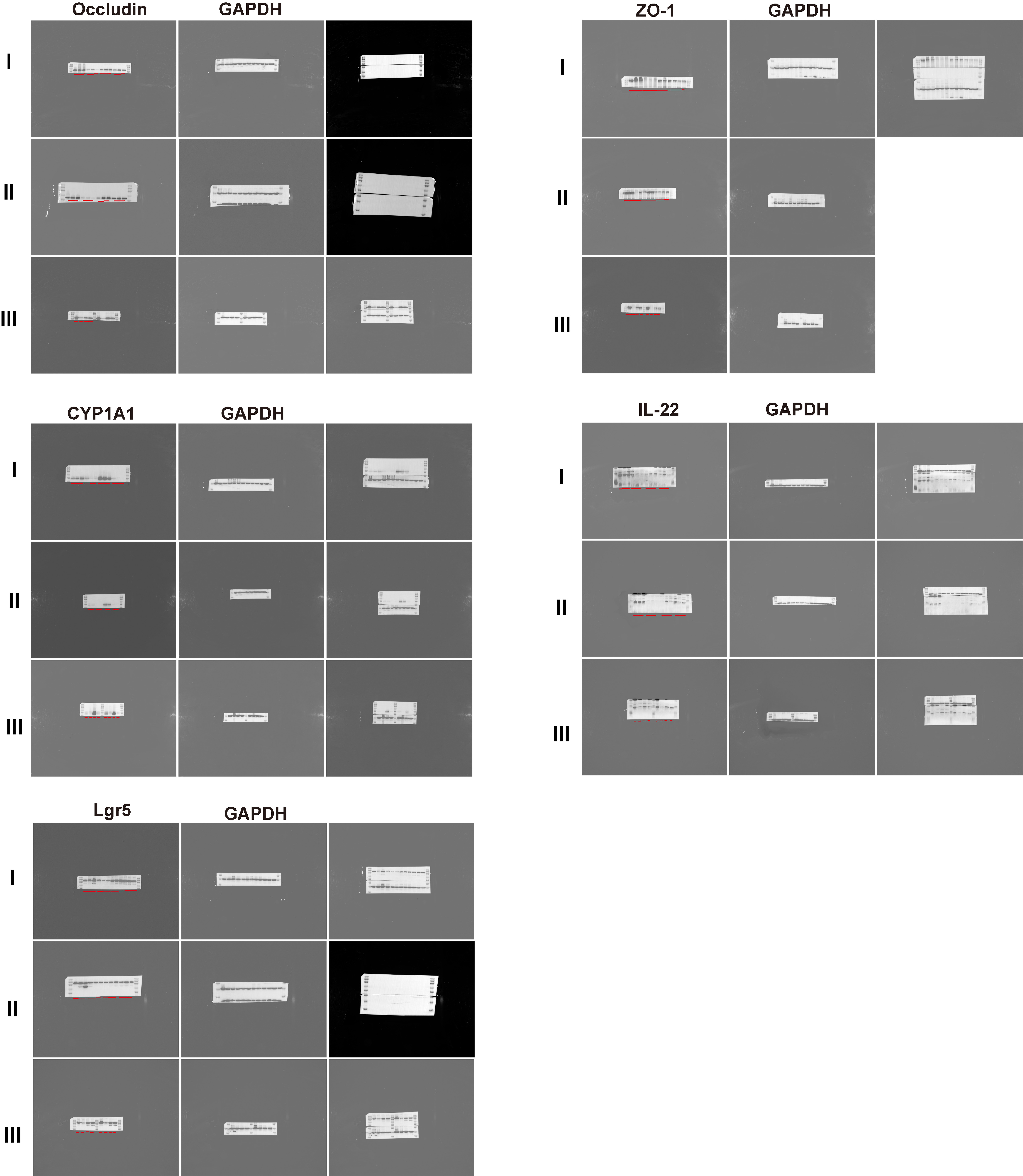

Supplement: S2 Fig — The original graph of the WB experiment, from left to right are Ctrl, DSS, DSS + HnAg, and DSS + SASP groups. (TIF) [file pntd.0012714.s002.tif]

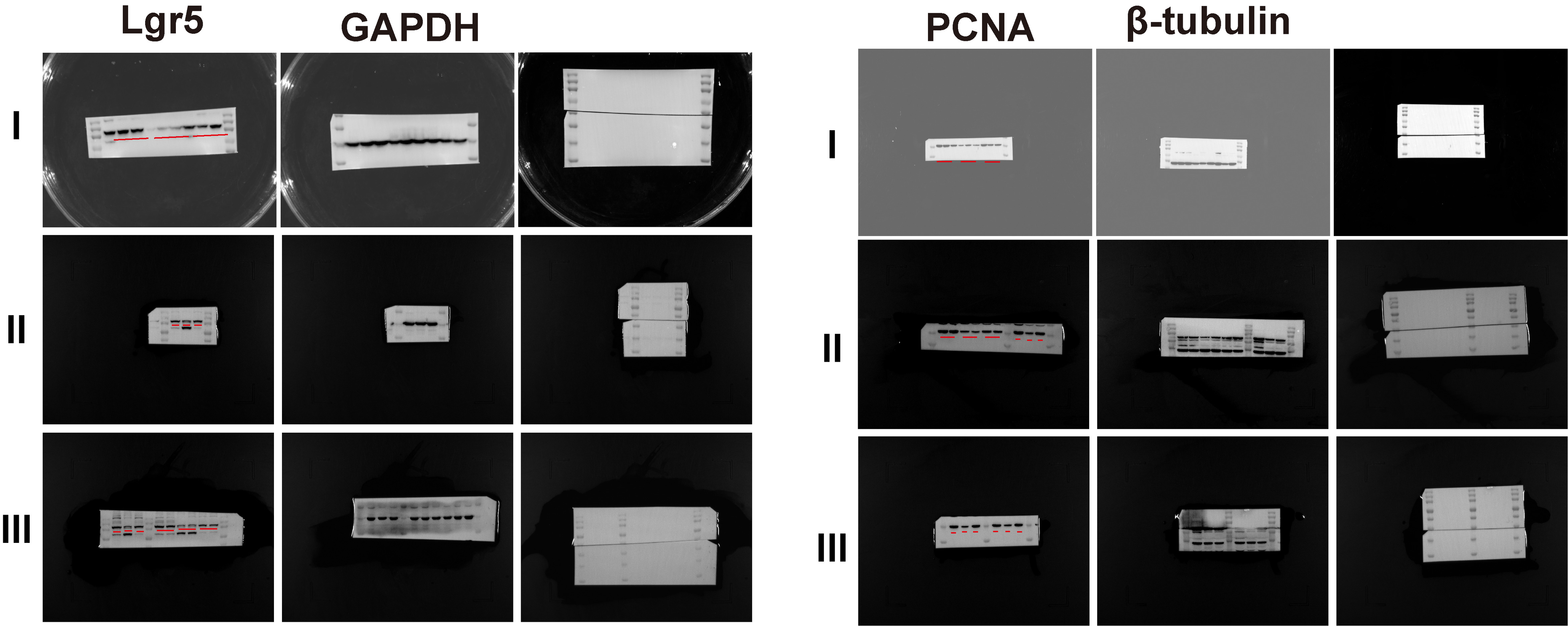

Supplement: S3 Fig — The data used for graphing in the manuscript, from left to right the Ctrl, DSS, and DSS + HnAg groups. (TIF) [file pntd.0012714.s003.tif]
