# Supplementary material for: Hymenolepis nana antigens alleviate ulcerative colitis by promoting intestinal stem cell proliferation and differentiation via AhR/IL-22 signaling pathway
Source: PLoS Negl Trop Dis. 2024 Dec 12;18(12):e0012714. doi: 10.1371/journal.pntd.0012714 (PMC11670978; doi:10.1371/journal.pntd.0012714)
Supplement: S1 Table — (DOCX) [file pntd.0012714.s004.docx]

**Table S1. Primer sequences used for RT-qPCR experiments of the current study**

| Target Genes | Primers Sequences (5’ - 3’) |
| --- | --- |
| *COX-I* | F: ACCGCGTCGTGTGTGTATTT  R: ACATGCAA CTGGGCTCATACG |
| *IL-6* | F: TGGGACTGATGCTGGTGAC  R: CACAACTCTTTTCTCATTTCCACG |
| *IL-1β* | F: AGCAGCTATGGCAACTGTTC  R: ACAGGTCATTCTCATCACTGTCAA |
| *TNF-α* | F: ACGGCATGGATCTCAAAG  R: TGGGAGTAGACAAGGTACAACC |
| *IFN-γ* | F: CACACCTGATTACTACCTTCTTCAG  R: GACTCCTTTTCCGCTTCCTGAGG |
| *IL-10* | F: ACCAATAGCTGATGTTGCCA  R: GAATGATGCCAGAGCTACGA |
| *Lgr5* | F: CCTGGGAAAGCATACCCGTT  R: GGTTGACTCACAGGACCGTT |
| *GAPDH* | F: AGGAGCGAGACCCCACTAACA  R: AGGGGGGCTAAGCAGTTGGT |
