# Supplementary material for: Hymenolepis nana antigens alleviate ulcerative colitis by promoting intestinal stem cell proliferation and differentiation via AhR/IL-22 signaling pathway
Source: PLoS Negl Trop Dis. 2024 Dec 12;18(12):e0012714. doi: 10.1371/journal.pntd.0012714 (PMC11670978; doi:10.1371/journal.pntd.0012714)
Supplement: S2 Table — (DOCX) [file pntd.0012714.s005.docx]

**S2 Table. Scoring system for disease activity index (DAI) in the mice**

| Loss of body weight (%) | Shape of feces | Bloody stools | Score |
| --- | --- | --- | --- |
| <1 | normal | — | 0 |
| 1-5 | loose | （+） | 1 |
| 5-10 | semi-formed loose stool | 2（+） | 2 |
| 10-15 | loose, not attached to the anus | 3（+） | 3 |
| ≥15 | loose, attached to the anus | 4（+） | 4 |
